# Predictive value of cerebral perfusion and guanine nucleotide-binding protein, alpha-stimulating activity polypeptide in ischemic white matter lesions: a machine learning approach

**DOI:** 10.3389/fneur.2025.1612379

**Published:** 2025-10-29

**Authors:** Ning Yu, Shuai Ma, Zongkai Wu, Zhijie Dou, Shengxian Jiao, Yajing Li, Hebo Wang, Xiaoxuan Zhang

**Affiliations:** ^1^Department of Neurology, Affiliated Hospital of Chengde Medical University, Chengde, Hebei, China; ^2^Hebei Key Laboratory of Never Injury and Repair, Chengde, Hebei, China; ^3^Institute of Traditional Chinese Medicine, Chengde Medical University, Chengde, Hebei, China; ^4^Hebei Province Key Laboratory of Traditional Chinese Medicine Research and Development, Chengde, Hebei, China; ^5^Department of Neurology, Hebei General Hospital, Shijiazhuang, Hebei, China

**Keywords:** cerebral perfusion, white matter lesion, guanine nucleotide-binding protein, alpha-stimulating activity polypeptide, arterial spin labeling

## Abstract

**Purpose:**

To assess the predictive value of guanine nucleotide-binding protein, alpha-stimulating activity polypeptide (GNAS) and cerebral perfusion in various vascular regions for the severity of ischemic white matter lesions (WMLs).

**Methods:**

Patients hospitalized at the Neurology Department of the Affiliated Hospital of Chengde Medical University between April and November 2023 were evaluated for ischemic cerebral WMLs using magnetic resonance imaging. In this retrospective cohort study, patients were classified into two groups: mild and severe, based on Fazekas scores. White matter perfusion was assessed using image segmentation of arterial spin labeling sequence images. Predictive variables were identified via machine learning (ML). GNAS levels in peripheral blood were measured to explore their association with WML severity.

**Results:**

Among 85 patients (43 mild [24 males and 19 females], 42 severe [27 males and 15 females]), significant differences were observed in age (64.00 ± 8.47 years vs. 68.38 ± 10.85 years, *p* = 0.041), cerebral atrophy (37.2% vs. 71.4%, *p* = 0.002), and history of hypertension (41.7% vs. 77.0%, *p* = 0.002). Corpus callosum perfusion was lower in the severe group (35.84 ± 6.34 vs. 31.73 ± 8.60 mL/[min·100 g], *p* = 0.037). ML yielded 77.27% model accuracy. Although no significant difference in GNAS levels was observed (*p* = 0.375), a significant difference was noted in the Fazekas scores (*p* < 0.001).

**Conclusion:**

In patients with ischemic WMLs, factors such as age, sex, history of cerebral infarction, GNAS levels, and specific perfusion metrics are predictive of WML progression. Advanced imaging and ML improve detection. GNAS levels correlated with Fazekas scores, indicating their downregulation in the hypoperfused white matter.

## 1 Introduction

With an aging population, cognitive impairment has emerged as a critical factor threatening quality of life and imposing significant social and economic burdens (1). Previous studies indicate that chronic cerebral hypoperfusion (CCH) (2), resulting from a long-term insufficient blood supply to brain tissue, leads to sustained damage to the nervous system and is a significant contributing factor to Alzheimer’s disease (3) (AD) and vascular dementia (2) (VD). Consequently, some researchers propose that CCH may serve as an early preclinical biomarker of cognitive impairment, particularly in relation to AD (4, 5). Due to the insidious onset of cognitive impairment, patients often miss significant opportunities for prevention, making the identification of early markers a significant research challenge.

In clinical practice, insufficient perfusion in white matter regions and hemodynamic changes can lead to white matter lesions (WMLs), which are common imaging markers. WMLs primarily appear in the white matter of the brain on T2-weighted imaging (T2 WI) and fluid-attenuated inversion recovery (FLAIR) sequences, while T1-weighted imaging (T1 WI) shows either isointensity or a low signal (6). Clinical studies have noted decreased cortical cerebral blood flow (CBF) in patients with vascular cognitive impairment compared with that in age-matched controls (7). Impaired cerebral vessels in the deep white matter are linked to hemodynamic ischemic injury, resulting in increased WMLs (8). However, the status and extent of the decreased perfusion in the white matter remain unreported. Further research is necessary to determine if a correlation exists between cortical CBF and white matter perfusion, whether they mutually influence each other or are causally linked, and whether they can be combined to enhance the identification of at-risk populations.

Machine learning (ML) is a framework and a set of methods that enable computers to simulate human learning, allowing systems to acquire knowledge, improve skills, and enhance performance. Through processes such as model training, prediction, regression, classification, and clustering analysis, research has been conducted on patients with WMLs (9). Some studies have utilized brain region segmentation to obtain the diffusion coefficients of various white matter fiber bundles, uncovering links between the microstructural integrity of distal nerve bundles, tract specificity, WMLs, and their potential connections to attention and executive function (10). However, no studies have specifically examined white matter perfusion. To address this gap, this study aimed to apply ML techniques to brain region segmentation, assess perfusion in specific areas, and analyze the relationship between white matter region perfusion and WMLs through modeling.

In a previous study, permanent ligation through bilateral typical carotid artery occlusion (BCCAO) in rats reliably induced white matter (WM) hypoperfusion, resulting in pathological changes, including white matter porosity, gliosis, and myelin loss, in the corpus callosum (CC) region (11). Through bioinformatics analysis, we discovered that the guanine nucleotide-binding protein alpha-stimulating activity polypeptide (GNAS) may be downregulated in ischemic and hypoxic environments. It acts as a transducer in various signaling pathways regulated by G protein-coupled receptors (GPCRs) and plays a key role in CCH-related lesions in the WM. Subsequent molecular experiments confirmed that GNAS expression decreased in the BCCAO group, accompanied by a corresponding decline in protein levels. Building on prior basic research and clinical applications, this study aimed to analyze the correlation between the severity of WMLs and the perfusion of white matter regions in patients with ischemic WMLs, while also investigating GNAS expression in this patient population.

## 2 Materials and methods

### 2.1 Ethics considerations

The study protocol was approved by the hospital’s ethics committee, and all patients or their guardians provided written informed consent. The study protocols had been reviewed by the Ethics Committee of the Affiliated Hospital of Chengde Medical University (CDMU; CYFYLL2023507).

### 2.2 Participants

This retrospective study included consecutive patients admitted to the Department of Neurology at the Affiliated Hospital of CDMU from October 2022 to April 2023. It aimed to improve the detection of ischemic WMLs using magnetic resonance imaging (MRI).

### 2.3 Inclusion criteria

(1)  Age 18 years or older.
(2)  Imaging diagnostic criteria for high signal intensity in ischemic brain WM confirmed by head MRI: Head MRI revealing paraventricular and deep WM or subcortical regions, with sheet-like lesions or diffuse T2-weighted imaging (T2 WI) changes, T2-FLAIR sequence, and T1-weighted imaging (T1 WI) showing equal or low signal intensity.
(3)  Arterial Spin Labeling technology (ASL).
(4)  Signed the informed consent form.

### 2.4 Exclusion criteria

(1)  History of trauma, tumor, systemic immune disease, poisoning, central nervous system infection, or autosomal dominant cerebral artery disease with subcortical infarction and leukoencephalopathy, except for high WM signals due to other causes.
(2)  History of cerebral hemorrhage, subarachnoid hemorrhage, or intracranial surgery.
(3)  History of acute infarction, cerebral hemorrhage, or transient ischemic attack (TIA).
(4)  A plain head MRI scan that was not performed simultaneously with ASL.

### 2.5 Demographic characteristics collection

Demographic characteristics, including sex, age, medical history (e.g., hypertension and diabetes), and lifestyle factors (e.g., smoking and alcohol consumption), were gathered from electronic medical records at the Affiliated Hospital of CDMU.

### 2.6 Imaging data

Cerebral MR images were obtained from the Picture Archiving and Communication System at the Affiliated Hospital of CDMU. The severity of WMLs was evaluated using FLAIR imaging. Individual CBF maps were created from each perfusion-weighted difference image derived from ASL.

#### 2.6.1 MR image acquisition

MR images for all enrolled patients were performed using a GE 3.0-T superconducting magnetic resonance machine (SIGNA Pioneer). The MRI protocol included 3D-ASL and routine sequences (e.g., pre−/post-contrast T1-weighted, pre−/post-contrast T2-weighted, diffusion-weighted imaging (DWI), and FLAIR images).

Specific scan parameters were as follows:

T1WI: TR: 1750 ms, TE: 8.4 ms, FOV: 24 mm × 24 mm, acquisition matrix: 320 mm × 224 mm, layer thickness: 5 mm, layer distance: 1 mm, scanning time: 1 min.
T2WI: TR: 7303 ms, TE: 106.8 ms, FOV: 24 mm × 24 mm, acquisition matrix: 352 × 352 mm, layer thickness: 5 mm, layer distance: 1 mm, scanning time: 44 s.
FLAIR: TR: 6500 ms, TE: 100.6 ms, FOV: 24 mm × 24 mm, acquisition matrix: 260 × 260 mm, layer thickness: 5 mm, layer distance: 1 mm, scanning time: 1 min 45 s.
DWI: TR: 3624 ms, TE: 86.2 ms, FOV: 24 mm × 24 mm, acquisition matrix: 128 × 128 mm, layer thickness: 5 mm, layer distance: 1 mm, scanning time: 42 s.
ASL: Post-labeling delays (PLD): 2.5 s, TR: 5344 ms, TE: 10.9 ms, acquisition matrix: 512 × 512 mm, FOV 24 mm × 24 mm, thickness: 4 mm, number of layers: 36, time: 4 min 49 s; PLD: 1.5 s, TR: 4649 ms. TE: 10.9 ms, acquisition matrix: 512 × 512 mm, FOV 24 mm × 24 mm, thickness: 4 mm, number of layers: 36, time: 4 min 15 s.

#### 2.6.2 WMLs’ severity definition and grading

The severity of WMLs was assessed using the Fazekas scale (12), which provides separate scores for paraventricular and deep WMLs. The specific criteria were as follows:

A: Paraventricular WMLs (periventricular lesions, PVLs):
0 – no lesion; 1 – cap or pencil-like thin-layer lesion; 2 – lesions with a smooth halo; and 3 – irregular paraventricular high signal extending into the deep WM.
B: Deep brain WML (deep white matter lesions, DWMLs) signal:
0 – no lesion; 1 – point-like strong lesion; 2 – lesion beginning to fuse; and 3 – large-area fused lesions.

The total score (A + B) was calculated as the WMLs severity score, with scores of 3 or more classified as the severe group and scores of 2 or less classified as the mild group.

#### 2.6.3 CBF determination and post-ASL treatment of WM fibers

Relevant images were obtained using the MR750W magnetic resonance system, and ASL was used to measure perfusion based on the raw images acquired with PLD of 1.5 s and 2.5 s. Quantitative data were automatically analyzed using the CereFlow software of Anyi (Beijing) Technology Co., Ltd. through the following steps: (a) CBF images were calculated from the original ASL images (13). (b) The MO images were registered to T1 space, and the ASL data were converted to T1 space using a 3D rigid transformation. (c) The MNI152 template was registered to T1 space, and the WM fiber tag map (ICBM-DTI-81) (14) was converted to T1 space. (d) The average CBF value was calculated for each region in the map. The corr-CBF represents the regional cerebral blood flow value after spatial registration and regional standardization extraction, which helps to reduce the variation caused by individual brain structure differences and thereby improve the accuracy of regional comparison.

### 2.7 Serum GNAS assay

All patients had 5 mL of fasting venous blood collected, left to stand at room temperature for 30 min, and then centrifuged at 1000 g for 15 min. The supernatant was aspirated and stored in a refrigerator at −80 °C. Serum GNAS level was measured using enzyme-linked immunosorbent assay (ELISA), following the manufacturer’s instructions (IAIBO [Wuhan] Science and Technology Co., Ltd., Product No.: E14288h).

Briefly, (a) Sample addition: Blank, standard, and sample wells were prepared. Except for the blank wells, 100 μL of the standard solution or sample was added to the remaining wells, mixed gently, and the microplate was covered with a lid and incubated at 37 °C for 120 min. (b) The liquid was discarded, and the samples were blotted dry. Then, 100 μL of detection solution A was added to each well, followed by gentle shaking and mixing. The microplate was then covered with a film and incubated at 37 °C for 60 min. (c) The liquid was discarded, the walls were blotted dry, and the plate was washed three times. Each well was soaked for 1–2 min with approximately 300 μL per well and then blotted dry. (d) Precisely 100 μL of detection solution B was added to each well, and the microplate was covered with a film and incubated at 37 °C for 60 min. The plate was then washed five times (as per step c). (e) Exactly 90 μL of substrate solution was sequentially added to each well, and color development was carried out at 37 °C in the dark for 15 min. (f) Exactly 50 μL of stop solution was sequentially added to each well to terminate the reaction. (g) The optical density of each well was measured sequentially at a wavelength of 450 nm using an ELISA reader.

### 2.8 Data processing

#### 2.8.1 Basic data processing

Employing SPSS version 25 statistical software, quantitative data that conform to the normal distribution and homogeneity of variance were expressed as mean±standard deviation. Independent sample *t*-tests were used to compare the two groups. When the assumptions were not met, data were presented as the median (P25, P75), and non-parametric Mann–Whitney tests were used for group comparisons. Qualitative data were presented as percentages (%), and chi-square tests were employed for intergroup comparisons. Kruskal–Wallis tests were applied for comparisons among multiple groups that did not follow a normal distribution, followed by Dunn’s multiple comparisons for pairwise comparisons. The significance level was set at *p*-values <0.05.

#### 2.8.2 Strategies related to deep learning

Using Python version 3.7.4, the collected data were systematically cleaned and organized. Participants were categorized into two groups based on the severity of WMLs: the mild group received a value of 0, while the severe group received a value of 1. To identify predictive indicators beyond the severity of WMLs, the SelectKBest and f_classif features from the sklearn. The feature_selection module was utilized for feature selection. Using SelectKBest (score_func = f_classif) to select the 10 features most correlated with WMLs severity based on Analysis of Variance *F*-values. The most significant factors were subsequently chosen to construct a new dataset. This new dataset was then divided into training and testing subsets using the train_test_split function, with a test size of 20% (test_size = 0.2) and a random state of 42 to ensure reproducibility. Furthermore, the hyperparameters of our random forest (n_estimators = 100, max_depth = 10) and the use of five-fold cross-validation were employed to prevent overfitting and enhance the model’s ability to generalize to new data. The accuracy of the predictive model was evaluated using the accuracy_score function. Furthermore, the model’s performance was validated against the test set, and a corresponding receiver operating characteristic (ROC) curve was generated to illustrate the model’s discriminative ability. Relevant software tools were employed for analysis.

## 3 Results

### 3.1 Baseline demographics of patients in both groups

**Figure 1:**
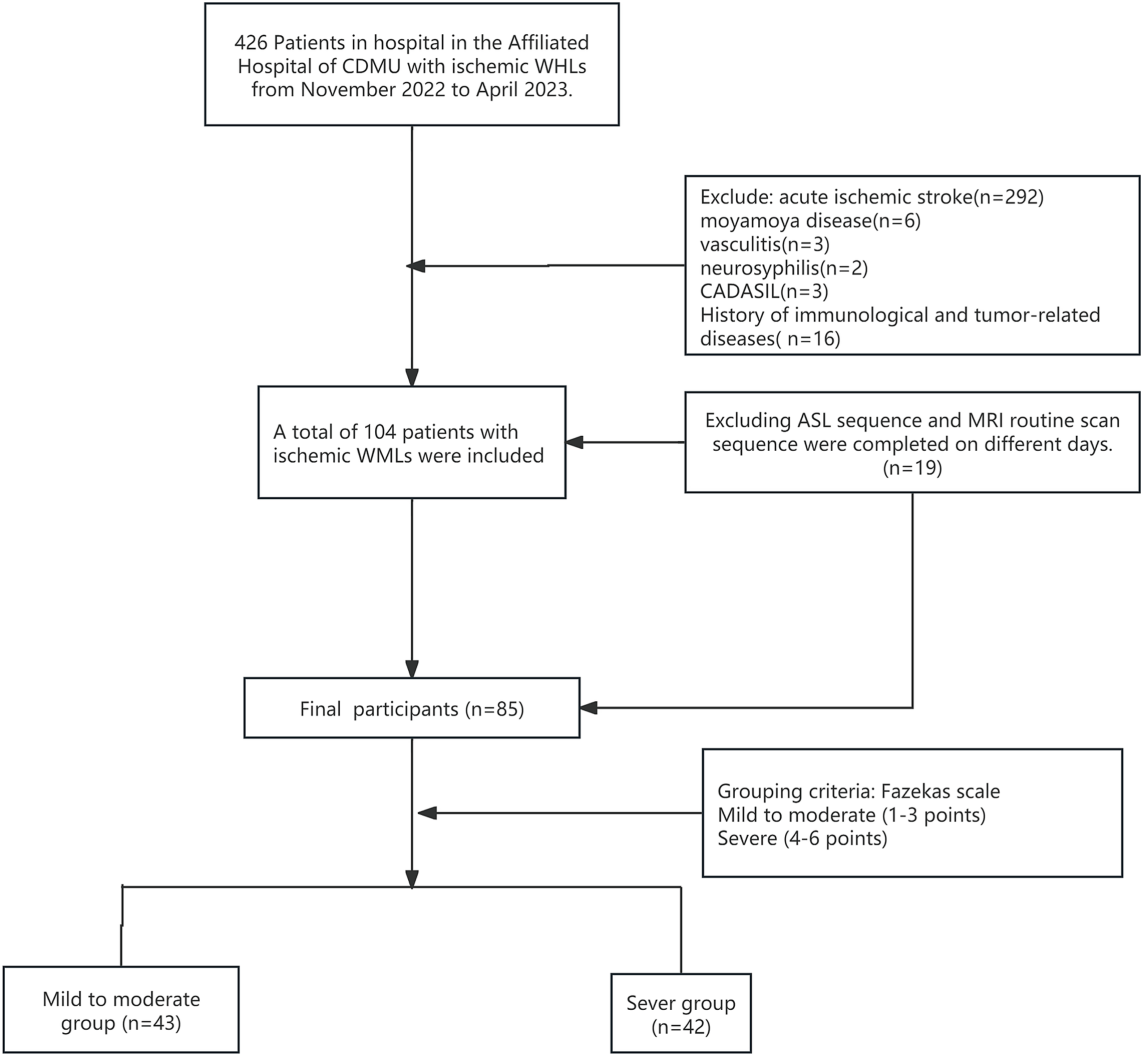
Final participants enrollment flow chart. CDMU, Chengde Medical University; CADASIL, cerebral autosomal dominant arteriopathy with subcortical infarcts and leukoencephalopathy; WMLs, white matter lesions.

During this period, 426 patients were diagnosed with ischemic leukodystrophy, and 104 of them met the inclusion criteria for the study. After excluding 19 patients who did not complete the relevant sequence on the same day due to the requirement of applying T1WI registration for segmenting the ASL sequence perfusion as outlined in the protocol, the final cohort consisted of 85 patients. Severity was categorized according to the Fazekas scale (Figure 1). The cohort consisted of 51 males and 34 females, aged 41–89 years (mean age: 66.16 ± 9.91 years). According to the Fazekas classification, the mild group consisted of 43 patients (24 males and 19 females), while the severe group included 42 patients (27 males and 15 females). The average age of all participants in the mild group was 64.00 ± 8.47 years, whereas the severe group had an average age of 68.38 ± 10.85 years, with a statistically significant difference observed (*p* = 0.041). The prevalence of brain atrophy was 37.2% in the mild group, compared to 71.4% in the severe group, reflecting a significant difference (*p* = 0.002). Additionally, a history of hypertension was present in 41.7% of the mild group and 77.0% of the severe group, showing a statistically significant difference (*p* = 0.002). However, no significant differences were found between the mild and severe groups regarding the history of cerebral infarction (62.5% in the mild group vs. 72.4% in the severe group), diabetes (16.7% in the mild group vs. 23.0% in the severe group), smoking history (33.3% in the mild group vs. 36.1% in the severe group), or alcohol consumption (29.2% in the mild group vs. 33.1% in the severe group), with all *p*-values exceeding 0.05 (see Table 1).

**Table 1:** Demographic baseline data table.

Index	Group	Mild to moderate group	Severe group	t/Z/χ^2^	*P*
Gender	M	24 (55.8%)	27 (64.3%)	0.635	0.425
	F	19 (44.2%)	15 (35.7%)		
Age		60.08 ± 7.67	68.56 ± 9.71	−3.826	0.041
Encephalatrophy	Deny	27 (62.8%)	12 (28.6%)	10.020	0.002
	Yes	16 (37.2%)	30 (71.4%)		
History of cerebral infarction	Deny	14 (32.6%)	10 (23.8%)	0.803	0.370
	Yes	29 (67.4%)	32 (76.2%)		
Hypertension	Deny	21 (48.8%)	7 (16.7%)	9.955	0.002
	Yes	22 (51.2%)	35 (83.3%)		
Diabetes	Deny	35 (81.4%)	32 (76.2%)	0.345	0.557
	Yes	8 (18.6%)	10 (23.8%)		
Drinking history	Deny	30 (69.8%)	29 (69.0%)	0.005	0.943
	Yes	13 (30.2%)	13 (31.0%)		
Smoking history	Deny	26 (60.5%)	29 (69.0%)	0.685	0.408
	Yes	17 (39.5%)	13 (31.0%)		

### 3.2 Analysis of WM in both the mild and severe groups

**Figure 2:**
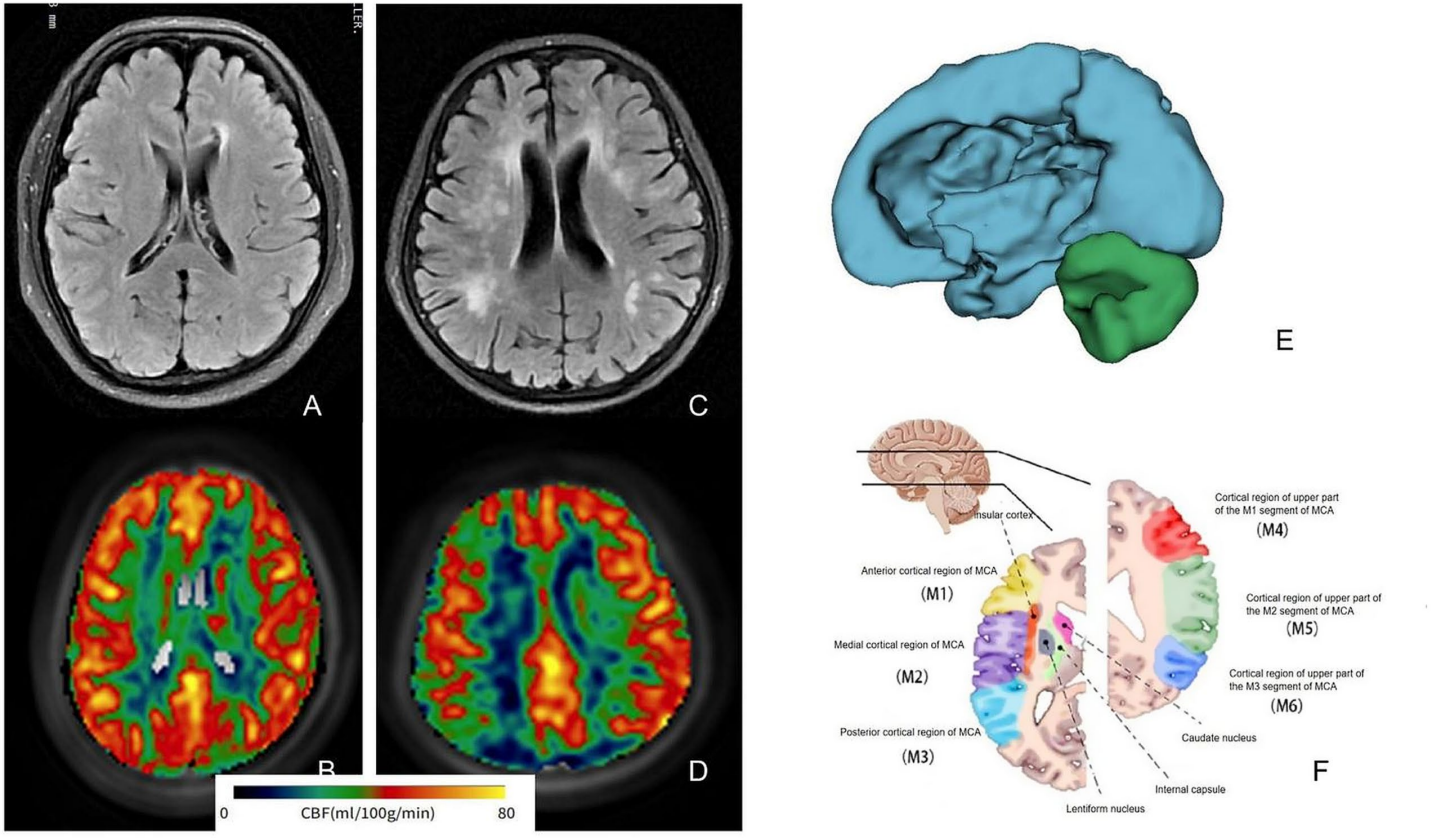
Magnetic resonance analysis of typical WMLs patients shows that **(A,B)** are FLAIR images and related pseudo-color images of mild patients, and **(C,D)** are related image data of severe patients. It can be seen that the perfusion of severe patients is relatively low. **(E)** Shows the process of brain region extraction, and **(F)** is the schematic image of brain region segmentation at this level. WMLs, white matter lesions; MCA, middle cerebral artery.

WM region markers were extracted and segmented following the registration of all raw T1WI data to a standard brain segmentation template using CerebralFlow, resulting in 48 anatomical regions. CBF was calculated for PLDs of 1.5 s and 2.5 s to align with the ASL data, with corrected CBF (corr-CBF) derived through appropriate adjustments (refer to Figure 2 for typical examples and registration). Perfusion in the CC was analyzed for both groups, yielding values of 35.84 ± 6.34 mL/(min·100 g) in the mild group and 31.73 ± 8.60 mL/(min·100 g) in the severe group. Additionally, CBF values in the column and fornix were 40.59 ± 8.83 mL/(min·100 g) and 34.28 ± 7.12 mL/(min·100 g), respectively, with *p*-values of 0.037 and 0.01. No significant differences were observed in perfusion across the other brain regions (*p* > 0.05; Table 2). Given that this study explored and compared multiple brain regions, the results should be regarded as preliminary findings. Further studies with larger sample sizes are needed to verify these results.

**Table 2:** Cerebral perfusion in each white matter region.

Index	Perfusion mL/(min*100 g)		*t*	*P*
	Mild to moderate	Severe		
Brachium pontis_corr-CBF	40.61 ± 7.57	39.79 ± 6.23	0.514	0.608
Fasciculus crossed pontis_corr-CBF	47.17 ± 10.58	44.88 ± 9.25	0.983	0.328
Knee of the corpus callosum_corr-CBF	33.84 ± 3.96	33.10 ± 6.30	0.536	0.593
Corpus callosum body_corr-CBF	35.84 ± 6.34	31.73 ± 8.60	2.121	0.037
Corporis callosi splenium_corr-CBF	34.85 ± 7.95	32.73 ± 8.45	1.060	0.292
Fornix corr-CBF	40.59 ± 8.83	34.28 ± 7.12	3.430	0.001
Corticospinal tract (R)corr-CBF	44.26 ± 9.57	41.86 ± 7.91	1.185	0.239
Corticospinal tract (L) corr-CBF	42.86 ± 7.59	41.31 ± 7.20	0.878	0.382
Lemniscus medialis (R) corr-CBF	41.54 ± 11.30	39.75 ± 8.73	0.781	0.437
Lemniscus medialis (L)corr-CBF	42.76 ± 11.17	41.00 ± 15.38	0.510	0.611
Corpora restiformia(R)_corr-CBF	43.60 ± 9.39	43.59 ± 16.66	0.004	0.997
Corpora restiformia(L)_corr-CBF	43.82 ± 8.10	41.75 ± 9.75	0.918	0.361
Pedunculus cerebellaris superior (R)_corr-CBF	41.34 ± 8.41	41.92 ± 10.48	−0.244	0.808
Pedunculus cerebellaris superior(L)_corr-CBF	40.78 ± 8.12	41.03 ± 11.30	−0.097	0.923
Pedunculus cerebri_(R)_corr-CBF	40.98 ± 6.03	40.70 ± 7.82	0.157	0.876
Pedunculus cerebri (L)_corr-CBF	40.54 ± 7.23	40.55 ± 5.93	−0.003	0.998
Anterior limb of internal capsule_(R)_corr-CBF	38.84 ± 5.94	40.89 ± 6.69	−1.312	0.193
Anterior limb of internal capsule_(L)_corr-CBF	41.27 ± 6.88	41.30 ± 6.59	−0.017	0.986
Posterior limb of internal capsule_(R)_corr-CBF	34.58 ± 5.50	36.25 ± 6.84	−1.065	0.290
Posterior limb of internal capsule_(L)_corr-CBF	34.34 ± 5.17	36.94 ± 6.20	−1.821	0.072
Posterior lens of internal capsule_(R)_corr-CBF	35.34 ± 6.52	36.48 ± 8.01	−0.620	0.537
Posterior lens of internal capsule_(L)_corr-CBF	37.79 ± 7.59	37.95 ± 8.07	−0.082	0.935
Anterior radiation crown_(R)_corr-CBF	29.21 ± 3.53	30.37 ± 6.28	−0.849	0.399
Anterior radiation crown_(L)_corr-CBF	30.21 ± 4.51	30.18 ± 6.55	0.022	0.982
Upper radiation crown_(R)_corr-CBF	27.45 ± 3.92	27.58 ± 6.46	−0.090	0.928
Upper radiation crown_(L)_corr-CBF	28.17 ± 4.18	27.95 ± 6.09	0.162	0.872
Posterior radiation crown_(R)_corr-CBF	26.88 ± 5.26	26.22 ± 6.81	0.428	0.669
Posterior radiation crown_(L)_corr-CBF	27.08 ± 4.79	26.25 ± 6.32	0.581	0.563
Posterior thalamic radiation (Including apparent radiation)_(R)_corr-CBF	27.48 ± 6.16	26.46 ± 8.48	0.533	0.595
Posterior thalamic radiation (Including apparent radiation)__(L)_corr-CBF	27.29 ± 5.69	26.63 ± 8.79	0.338	0.736
Sagittal layer (including inferior longitudinal fasciculus and inferior fronto-occipital fasciculus)(R)_corr-CBF	35.22 ± 5.90	34.17 ± 7.11	0.641	0.523
Sagittal layer (including inferior longitudinal fasciculus and inferior fronto-occipital fasciculus)__(L)_corr-CBF	37.03 ± 7.32	35.05 ± 6.72	1.187	0.238
Capsula externa_(R)_corr-CBF	38.69 ± 6.41	39.25 ± 7.06	−0.335	0.738
Capsula externa_(L)_corr-CBF	40.76 ± 7.47	39.23 ± 6.38	0.950	0.345
Cingulate gyrus_(R)corr-CBF	46.65 ± 9.73	43.23 ± 9.56	1.476	0.144
Cingulate gyrus (L)_corr-CBF	46.19 ± 8.84	42.96 ± 8.69	1.533	0.129
Cingulate (hippocampus) (R)_corr-CBF	45.77 ± 8.31	44.90 ± 8.33	0.433	0.666
Cingulate (hippocampus) (L)_corr-CBF	45.76 ± 8.42	45.46 ± 6.93	0.169	0.866
Fornix/striae terminalis_(R)_corr-CBF	45.25 ± 8.93	41.17 ± 8.38	1.987	0.050
Fornix/striae terminalis (L)_corr-CBF	44.23 ± 9.67	42.14 ± 8.47	0.984	0.328
Fasciculus arcuatus (R)corr-CBF	34.98 ± 7.78	34.07 ± 7.49	0.502	0.617
Fasciculus arcuatus (L)_corr-CBF	36.00 ± 7.86	33.97 ± 6.82	1.183	0.240
Fasciculus fronto-occipitalis supratica _(R)_corr-CBF	29.86 ± 5.84	32.10 ± 5.99	−1.560	0.122
Fasciculus fronto-occipitalis supratica (L)_corr-CBF	31.80 ± 5.05	31.86 ± 5.99	−0.042	0.966
Unciform fasciculus (R)_corr-CBF	32.54 ± 5.68	34.74 ± 7.53	−1.294	0.199
Unciform fasciculus_(L)_corr-CBF	34.17 ± 5.65	34.87 ± 6.14	−0.482	0.631
Tapetum_(R)_corr-CBF	24.38 ± 5.47	22.94 ± 8.78	0.743	0.459
Tapetum_(L)_corr-CBF	23.20 ± 5.20	22.99 ± 8.97	0.106	0.916

### 3.3 Results related to deep learning

Given the extensive number of brain areas examined, this analysis aimed to assess a broad range of variables. The SelectKBest package was utilized to screen the included factors. Since the perfusion data from the brain regions were continuously and normally distributed, variance-based feature screening was employed for the selection process (f_classif). The top 10 factors identified included sex, age, a history of cerebral infarction, GNAS level, CBF in the CC knee, CBF in the body of the CC, CBF in the splenium of the CC, CBF in the upper radiation crown, CBF in the rear radiation crown, and CBF in the callosal crown.

**Figure 3:**
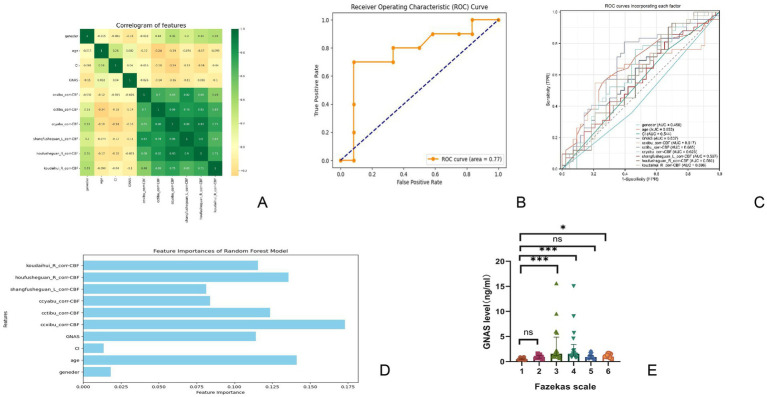
Data analysis situation **(A)** shows correlation heat map for screening relevant factors, **(B)** shows ROC curve of all-cause prediction model in predicting the severity of ischemic white matter lesions. **(C)** Shows ROC curve of the severity of leukin disease predicted by each predictor in this prediction model. **(D)** Shows prediction weights of selected factors. The vertical axis is the selected factors, and the horizontal axis is the weight of each factor. **(E)** Shows the distribution of GNAS in different Fazekas scores. GNAS, guanine nucleotide-binding protein, alpha-stimulating activity polypeptide.

Correlations among all the selected factors were analyzed, with a heat map revealing significant associations between these factors and perfusion in each brain region (Figure 3A). The selected factors were subsequently used to train a new dataset, resulting in an evaluation accuracy of 77.27% for the model. Feature weights were derived through weight analysis and were visualized using a bar chart (Figure 3D). The top five factors that can explain serious risk and their weight were as follows: the CBF in the CC knee (17.30%), age (14.13%), CBF in the posterior radiative crown (13.57%), CBF in the CC body (12.35%), and GNAS level (11.42%). The model’s overall performance, developed using the random forest algorithm, was evaluated using a ROC curve (Figure 3B). The area under the curve for the model was calculated to be 0.77, with a sensitivity of 70.00% and specificity of 83.33%. The confusion matrices are presented in Table 3. ROC curve analyses were also conducted for individual diagnostic factors (Figure 3C).

**Table 3:** Confusion matrices.

The actual situation	Prediction results	
	True	False
True	10	2
False	3	7

### 3.4 GNAS expression in each group

The GNAS levels for Fazekas score groups 1–6 were as follows: 0.52 ± 0.19 ng/mL, 20.95 ± 0.35 ng/mL, 31.45 (1.21, 2.16) ng/ml, 41.56 (1.18, 2.26) ng/ml, 50.92 (0.65, 1.18) ng/ml, and 61.16 ± 0.38 ng/mL. Additional analysis of specific scores revealed statistically significant differences in GNAS levels across the scoring groups (*p* < 0.0001, Z = 41.23). Further analysis revealed significant differences in GNAS levels between Fazekas group 1 and scores of 3, 4, and 6 points, as well as between 3 and 5 points (*p* < 0.05). No significant differences were observed among the other groups (Figure 3E).

## 4 Discussion

As a common neuroimaging finding and predictor of neurological dysfunction (15, 16), WMLs progress under complex mechanisms involving chronic hypoperfusion and oxidative stress, which disrupt oligodendrocyte function and remyelination (17–21). However, the specific role of regional perfusion patterns remains unclear. Our study, therefore, aimed to assess the predictive value of cerebral perfusion and the hypoxia-related biomarker GNAS for the severity of WML.

Our findings highlight specific factors associated with WMLs progression. While conventional vascular risks such as hypertension, diabetes, and smoking were not significant, patient age and sex were essential predictors, consistent with prior research (22). Moreover, disease progression was marked by volume loss in critical structures such as the corpus callosum and cingulate gyrus. This can be attributed to the pivotal role of regional cerebral hypoperfusion, which has been established as an independent predictor of white matter load in cerebral small vessel disease (23). Furthermore, deep WMLs (DWMLs) exhibited more severe perfusion deficits and a more substantial prognostic impact than periventricular WMLs (PVLs), likely reflecting their distinct pathogenic mechanisms. PVLs may arise from ependymal layer disruption (24), whereas DWMLs are linked to angiogenic dysregulation in deep small vessels (25), a pattern consistent with chronic hypoperfusion-induced structural damage in animal models (11). The strong association between CC hypoperfusion and the severity of WMLs highlights the vulnerability of this central white matter structure. The CC, as the major commissural pathway, is essential for interhemispheric communication, and its integrity is increasingly recognized as a marker of cerebrovascular health (26–28). Similarly, a community-based study revealed that microstructural alterations in WM regions, including the CC, corona radiata, subfrontal cortex, and cingulate gyrus, adversely affect health perceptions among participants (29).

Based on prior evidence that GNAS may regulate apoptosis in WM (11), we evaluated its potential as a biomarker for the progression of WMLs. However, serum GNAS levels in patients showed a nonlinear relationship with Fazekas scores—unlike animal models—with significant elevations specifically in Fazekas 3–4 subgroups. This discrepancy may be explained by GNAS’s primarily intracellular localization and role in GPCR signaling (30), making serum levels an indirect measure of its activity. Notably, outliers in Fazekas 3–4 may reflect blood–brain barrier disruption (31), which allows for extracellular leakage. These findings highlight the complexity of translating GNAS findings to clinical settings.

The application of ML in our study was motivated by the need to address the high-dimensional and potentially multicollinear nature of our dataset, which included perfusion values from numerous brain regions, GNAS levels, and clinical variables. Traditional statistical methods are limited in handling such complex variable interactions. ML, specifically Random Forest, not only manages these challenges but also provides two key advantages: it quantifies the relative importance of each predictor, revealing that corpus callosum perfusion was a more substantial contributor than age or hypertension; and it integrates all data types into a single, high-performance predictive model (AUC = 0.77). The new model incorporated more factors, indicating that the integrated model, which combines cerebral perfusion, GNAS, and clinical variables, has good discriminative power. Feature importance analysis further reveals that CBF in the knee of the corpus callosum is the most critical predictor of WML severity, with its contribution even exceeding that of age. This finding underscores the clinical significance of focusing on the perfusion of specific white matter pathways, which is not readily apparent in traditional univariate analyses.

In conclusion, in populations with WMLs, factors such as sex, age, history of cerebral infarction, GNAS levels, and CBF in the CC are significant contributors to the development of WMLs. Notably, hypoperfusion in the CC and the area of the corona radiata may exacerbate WMLs, affecting patients’ quality of life and warranting greater clinical attention. GNAS demonstrates predictive value in populations with high signal intensity, providing a more accurate interpretation of predictive outcomes. Additionally, regarding the distribution of scores, GNAS concentrations are notably elevated in individuals with Fazekas scores of 3 and 4, although this population also has a higher proportion of outliers. However, our study had several limitations. Its retrospective design and modest sample size limit the generalizability of the findings and increase the risk of overfitting in the machine learning model. The absence of health controls and standardized assessment of cognitive impairment due to various real-world problems directly prevents us from correlating our imaging and molecular findings with clinical functional outcomes. Future prospective studies with larger cohorts, including age-matched controls and comprehensive neuropsychological testing, are needed to validate these preliminary findings and clarify the role of CCH and GNAS in cognitive decline.

## Data Availability

The original contributions presented in the study are included in the article/supplementary material, further inquiries can be directed to the corresponding author.
